# Psychotherapy of Biases in Cognition in Schizophrenia: the SlowMo Randomised Controlled Trial for Paranoia, Outcomes and Mechanisms

**DOI:** 10.1192/j.eurpsy.2022.75

**Published:** 2022-09-01

**Authors:** P. Garety, T. Ward, R. Emsley, K. Greenwood, A. Hardy

**Affiliations:** 1 King’s College London, Psychology, London, United Kingdom; 2 King’s College London, Biostatistics, London, United Kingdom; 3 University of Sussex, Psychology, Brighton, United Kingdom

**Keywords:** Paranoia, Reasoning biases, Blended digital therapeutic, fast and slow thinking

## Abstract

Reasoning biases, specifically jumping to conclusions and belief inflexibility, may play a causal role in persistent paranoia. SlowMo, a new digitally supported blended cognitive-behavioural therapy, targets these biases. Adopting the terms ‘fast’ and ‘slow thinking’ as a heuristic to support therapy, SlowMo encourages people to notice a tendency to fast thinking, and to slow down for a moment to reduce paranoia. SlowMo therapy is the first digital blended therapy for paranoia, employing face to face therapy sessions with interactive digital content, and using mobile technology to promote generalisation to daily life. We report a randomised controlled trial with N=362 participants with distressing and persistent (3+months) paranoia, comparing 8 sessions of SlowMo plus Treatment as Usual (TAU) with TAU alone. We examined SlowMo’s effectiveness in reducing paranoia and improving reasoning biases; its mechanisms of action; usability; and acceptability (Garety et al., 2021). Outcomes: SlowMo was beneficial for paranoia: 10 /11 paranoia measures at 12 weeks and 8/11 at 24 weeks demonstrated significant effects, and sustained moderate effects were observed on all observer-rated measures of persecutory delusions. Improvements in self-esteem, worry, wellbeing and quality of life were also reported. Mediation: Consistent with the theory-driven design and treatment rationale, improvements in slower thinking were found to mediate change in paranoia at 12- and 24-week follow-ups. However contrary to hypothesis, reduced fast thinking did not mediate change in paranoia, whereas worry did. These findings highlight the potential therapeutic mechanisms of action of SlowMo which which are discussed further. Garety P, Ward T, Emsley R, et al. Effects of SlowMo, a Blended Digital Therapy Targeting Reasoning, on Paranoia Among People With Psychosis: A Randomized Clinical Trial. JAMA Psychiatry. 2021;78(7):714–725. doi:10.1001/jamapsychiatry.2021.0326

**Disclosure:**

No significant relationships.

